# The County Council as a Health Authority

**Published:** 1907-04-20

**Authors:** 


### THE COUNTY COUNCIL AS A HEALTH AUTHORITY.

### LIFE AND DEATH IN THE DOSS-HOUSE.

In the year 1894 the County Council first took over the oversight of common lodging-houses. The Council inspectors visit these by day and night, and prosecute owners who do not comply with the regulations of the Common Lodging-House Acts. Incidentally the Council accumulates a great deal of information which should be useful to those who really want to know how the poor live, and not merely to talk about it. First it appears that beds, either very cheap or absolutely free, exist in sufficient numbers to meet all the requirements of the population. The authorised accommodation in these lodging-houses has varied but little for a considerable number of years. In 1897 there was accommodation for 28,929 persons, in 1905 for 28,965, but within the intervening years it had been somewhat smaller, the lowest amount being in June 1902, when it stood at 28,441. And at no time during this period has the entire accommodation provided been made use of, though doubtless there was pressure in some localities while there were empty beds in others. But taking the metropolis as a whole, there has always been a considerable margin of empty beds. The highest number of persons recorded is at a census taken on November 14, 1903, when 25,107 slept in lodging-houses, but when a census was taken during 1904 and 1905 the number of occupants was on each occasion under 23,500. Yet withal, either from sheer poverty or from faults in the distribution of the available accommodation, there are always about 2,000 homeless vagrants wandering about the streets at night, or sleeping as best they can on staircases, in doorways, or under arches. An attempt made on the night of January 29, 1904, to make a census of these showed that the council inspectors counted in all 1,797 of these homeless vagrants, and a similar attempt made on February 17, 1905, gave a total of 2,181. That is to say, about one in 2,000 of the inhabitants of London have nowhere to lay their heads. It does not follow, however, that those who wander about all night have been similarly wandering all day. It is a general custom for the keepers of common lodging-houses to allow the frequenters of them to lounge about the kitchens all day, and, indeed, until closing time, which is between midnight and one o'clock in the morning. Until the house closes the keeper cannot tell who is and who is not going to pay for a bed, so they wait till the last moment before turning out the non-payers. Thus the night bird may have been dosing all day in a warm, if not absolutely comfortable kitchen. Of late some lodging-house keepers have abandoned this practice, and have allowed no one the shelter of the house who has not the means to pay for his bed; after this change it is reported that "those who before used to be 'turn-outs' found work and paid for their night's lodging." Whether or not London is the loadstone it is supposed to be to the countrymen, it seems certain that it is to its own children that the great city is most unkind. One of the County Council inspectors asked 318 persons who were receiving free food from the Church Army or the Salvation Army, where they came from, and the replies showed that 277 were Londoners, 39 came from the provinces, and only two were foreigners. The statistics of homelessness have a clear bearing upon the waves of unemployment, and we must infer that within the last two years trade has so far improved as to lift a considerable number of people from the level of the casual shelter of the lodging-house into something more permanent. We infer this from the fact that, though on the night of the census in February 1905 the Church Army had issued tickets for beds in lodging-houses to 1,600 men, the actual number of persons who slept in these lodging-houses was 60 less than at the census of the previous year.

As may well be imagined, the people who sleep in common lodging-houses are neither strong nor healthy. The most respectable among them are for the most part unskilled labourers, whom physical weakness renders uncertain of obtaining work—the men who are taken on only when better men cannot be got. But, thanks no doubt to the fact that they are under such strict supervision, these lodging-houses are not the breeding-places of infectious disease that some people think. The total number of notifiable infectious diseases reported in common lodging-houses in 1905 was 14—the lowest total since the Council had the administration of the Acts dealing with these places. This total was made up as follows: Erysipelas, 7 cases; scarlet fever, 5; diphtheria, 1; and enteric fever, 1. Yet the death-rate in common lodging-houses is high, much higher than in London as a whole. And the chief cause of death is phthisis. In the average death-rate phthisis follows an age-curve. In the earlier stages of adult life the death-rate from it is low. In young men between 25 and 35 of all classes the rate is 2.47 per 1,000; in the next decade it rises to 4.12 per 1,000, and between 45 and 55 reaches its maximum of 5.04 per 1,000. Between 55 and 65 it falls again to 4.20, and over 65 to 2.13 per 1,000. But in the common lodging-house phthisis is fatal in an ascending line. Even in the earlier years it works more mischief there than among the mass of men, killing 6.22 per 1,000 between the ages of 25 and 35, in the next decade 10.02 per 1,000, in the next 18.84, in the next 19.83, while in those over 65 the mortality from it is set down as 21.16. Among the old men of the lodging-houses, however, the most fatal disease is bronchitis, which kills 50.12 per 1,000, as against 16.36 of that age among the population as a whole. The death-rate approaches most nearly to the normal in diseases of the circulatory system, and the statistics show that between 55 and 65 the ordinary mortality is rather higher than that of the common lodging-house, namely, 8.47 as against 8.39, but over 65 the ordinary mortality is 22.07, while that of the lodging-house is 26.73. It will surprise many to learn that alcoholism is not responsible for a great proportion of deaths, even though, in the tables, it is reckoned to include cirrhosis of the liver. Deaths from this cause are highest among the oldest men, but even then they reach only 2.23 per 1,000—rather more than double the mortality from the same cause among the general male population, it is true, but by no means such a large proportion as one might expect from the character generally assigned to the "dosser." Among the unfortunates who find the lodging-house the best home they can procure, are many decent enough men, who are, however, too stupid or too weakly to raise themselves above its dreary level. They are unskilled, and when the unskilled labourer begins to lose his strength he loses all.

The bulk of the frequenters of these lodging-houses are men, but the authorised accommodation includes also beds for 2,340.5 women—the half being equivalent to one child. Of these beds doubtless a good number are actually occupied by children. A lodging-house is not much of a home for them, but it is better than walking the streets. The same may be said of the accommodation for 308 married couples, but one cannot help noting with satisfaction that the popularity of this department seems to be decreasing. Still, there can be no doubt that the common lodging-house, unattractive as it would seem to most of us, is a necessity of our time, and one can only be glad that it is under the competent supervision of the County Council.

